# Characterization of Ionotropic Receptor Gene *EonuIR25a* in the Tea Green Leafhopper, *Empoasca onukii* Matsuda

**DOI:** 10.3390/plants12102034

**Published:** 2023-05-19

**Authors:** Ruirui Zhang, Xiaoyue Lun, Yu Zhang, Yunhe Zhao, Xiuxiu Xu, Zhengqun Zhang

**Affiliations:** 1 College of Horticulture Science and Engineering, Shandong Agricultural University, Tai’an 271000, China; zhangruirui7275@163.com (R.Z.);; 2 Tea Research Institute, Shandong Academy of Agricultural Science, Ji’nan 250100, China

**Keywords:** *Empoasca onukii* Matsuda, *EonuIR25a*, plant volatiles, RNA interference, olfactory system

## Abstract

Ionotropic receptors (IRs) play a central role in detecting chemosensory information from the environment and guiding insect behaviors and are potential target genes for pest control. *Empoasca onukii* Matsuda is a major pest of the tea plant *Camellia sinensis* (L.) O. Ktze, and seriously influences tea yields and quality. In this study, the ionotropic receptor gene *EonuIR25a* in *E. onukii* was cloned, and the expression pattern of *EonuIR25a* was detected in various tissues. Behavioral responses of *E. onukii* to volatile compounds emitted by tea plants were determined using olfactometer bioassay and field trials. To further explore the function of *EonuIR25a* in olfactory recognition of compounds, RNA interference (RNAi) of *EonuIR25a* was carried out by ingestion of in vitro synthesized dsRNAs. The coding sequence (CDS) length of *EonuIR25a* was 1266 bp and it encoded a 48.87 kD protein. *EonuIR25a* was enriched in the antennae of *E. onukii*. *E. onukii* was more significantly attracted by 1-phenylethanol at a concentration of 100 µL/mL. Feeding with dsEonuIR25a significantly downregulated the expression level of *EonuIR25a*, after 3 h of treatment, which disturbed the behavioral responses of *E. onukii* to 1-phenylethanol at a concentration of 100 µL/mL. The response rate of *E. onukii* to 1-phenylethanol was significantly decreased after dsEonuIR25a treatment for 12 h. In summary, the ionotropic receptor gene *EonuIR25a* was highly expressed in the antennae of *E. onukii* and was involved in olfactory recognition of the tea plant volatile 1-phenylethanol. The present study may help us to use the ionotropic receptor gene as a target for the behavioral manipulation of *E. onukii* in the future.

## 1. Introduction

Insect chemoreception systems play a crucial role in many aspects of insect behaviors, such as foraging, mate recognition, oviposition, and predator avoidance [1,2]. The chemosensory system of insects plays an important role in insect and ecological niche adaptation and population evolution [3]. Chemosensory receptor proteins are normally expressed in the dendrites of chemosensory neurons and are specifically responsible for the interaction with chemosensory signals [4,5]. Olfaction is a highly specific, intricate, and extraordinarily sensitive system, and in the insect olfactory perception process, various olfactory proteins containing odorant binding proteins (OBPs), chemosensory proteins (CSPs), odorant receptors (ORs), ionotropic receptors (IRs), and odorant degrading enzymes are involved [6,7]. A large and highly divergent family of ionotropic glutamate receptor (iGluR)-related genes, called ionotropic receptors, has been screened and identified [8]. Four protein structural domains are covered in the structure of iGluR, including the amino-terminal domain (ATD), ligand binding domain (LBD), transmembrane domain (TMD), and carboxy-terminal domain (CTD) [9]. The extracellular ATD structural domain is followed by the LBD structural domain, which contains two semi-structural domains, S1 and S2 [10]. In the primary structure, S1 and S2 are separated by ion channel pores. The ion channel pore is formed by two transmembrane segments (TM1 and TM2) and a folded-in pore loop [11]. S2 is followed by a third transmembrane segment (TM3) and the cytoplasmic carboxy-terminal structural domain (CTD). The ATD, LBD, TMD, CTD, and coreceptor extra loop (CREL) are contained in coreceptors IR8a and IR25a [8,12].

IR25a is a highly conserved gene that evolved from a bilaterian non-NMDA receptor gene [12]. IR25a is expressed in different insect tissues, such as adult tentacles [13], proboscis [14,15], legs, wings, and abdomen [16]. IRs are involved in processes such as olfaction, taste sensation, hygrosensation, and cold sensation in insects [17,18]. The function of IRs requires co-expression of ubiquitous co-receptors, such as IR8a, IR25a, and IR76b [19,20]. *Drosophila* IR25a, as a co-receptor, was co-expressed with IR93a as well as IR40a in adult tentacles and is able to sense humidity in the environment [21]. In the arbovirus vector *Aedes aegypti*, *AeIR8a* null mutants lost neuronal and behavioral responses to acids [22]. RNAi knockdown of *AcIR76b* specifically impacts larval responses to butylamine in *Anopheles coluzzii* [23]. In *Mythimna separata*, *MsepIR8a* is a possible acid coreceptor among the putative acid-sensing IRs characterized [24]. In *Manduca sexta*, IR8a is essential for hexanoic acid- and 3-methylpentanoic acid-mediated fecal avoidance [25]. IR41a is sufficient to confer sensitivity to amine/imine compounds in *Anopheles gambiae* [26]. IR25a and IR76b are able to synergistically mediate the response to flavoring agents, including acetic acid, citric acid, tartaric acid, and hydrochloric acid therein [27]. In addition, IR25a is expressed in neurons of *Drosophila* larvae and adults to support thermosensation and wet sensation [21,28,29]. The study of the molecular mechanisms and functions of multiple sensory modalities of IR in insects will thus contribute to the use of olfactory mechanisms for efficient control of pests.

The tea plant *Camellia sinensis* (L.) O. Kuntze, is a significant cash crop in Asian countries, such as China, India, and Sri Lanka. The tender tea buds and leaves are usually plucked to produce high-grade tea as a beverage [30]. However, *C. sinensis* suffers heavily from attacks by many herbivorous insects in their life cycles. The tea green leafhopper, *Empoasca (Matsumurasca) onukii* Matsuda, an extremely harmful piercing pest with ten generations per year, is by far the gravest threat to tea plant cultivation [31]. Both nymphs and adults of *E. onukii* attack tea plants by using their piercing mouthparts (stylet), which ultimately results in plant yellowing, browning, and drying [32]. The most common methods used to control *E. onukii* are regularly applying chemical insecticides. However, the excessive use of pesticides is jeopardizing both human health and the environment. Organisms have genetically evolved to diversify to accommodate their environment [33]. Insects on tea plants could recognize odor molecules through chemosensory processs [34]. In *E. onukii*, *EonuCSP4* and *EonuCSP6-1* have binding affinities for farnesene, ocimene, and benzaldehyde, suggesting that they may be involved in chemosensory processes [35]. High expression levels of OBPs or CSPs in the head of *E. onukii* may be significant for sensing volatiles released by plants [36]. 13 *EonuCSPs* were highly expressed in the antennae, speculating they could participate in insect chemoreception [37], therefore, the exploration of chemosensory genes of insect pests on tea plants could be used for insect control strategies.

In this study, the ionotropic receptor gene *EonuIR25a* in *E. onukii* was cloned and bioinformatically analyzed. To characterize the *EonuIR25a* gene, sequence alignment and phylogenetic analysis were investigated. The expression levels of the *EonuIR25a* gene in different tissues of *E. onukii* were determined by quantitative real-time PCR (qRT-PCR). Behavioral responses of *E. onukii* to volatile compounds emitted by tea plants were determined by the olfactometer bioassay and field trials. To further explore the function of *EonuIR25a* in olfactory recognition of these compounds, RNAi of *EonuIR25a* was carried out by feeding with in vitro synthesized double-stranded RNA (dsRNA). This study provides the first comprehensive characterization of the *EonuIR25a* gene from *E. onukii.* Our findings should provide valuable insights into the design and implementation of novel strategies to control the damage caused by this tea plants pest.

## 2. Results

### 2.1. Sequence Analysis of the EonuIR25a Gene

**Figure 1:**
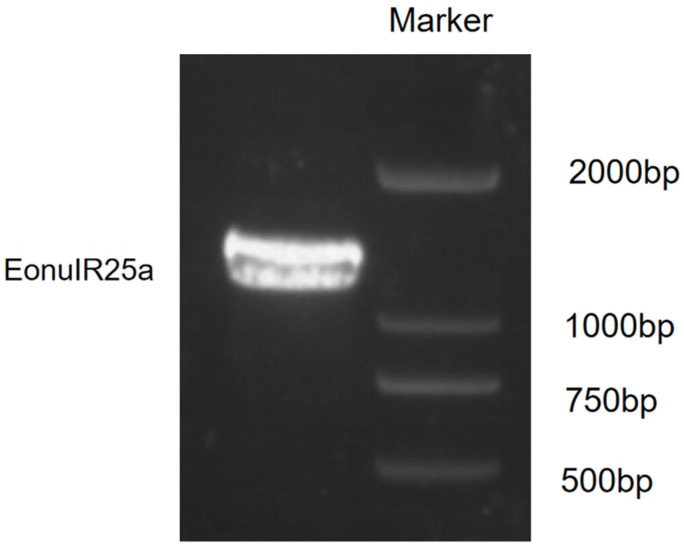
Identification of PCR products of *EonuIR25a*.

**Figure 2:**
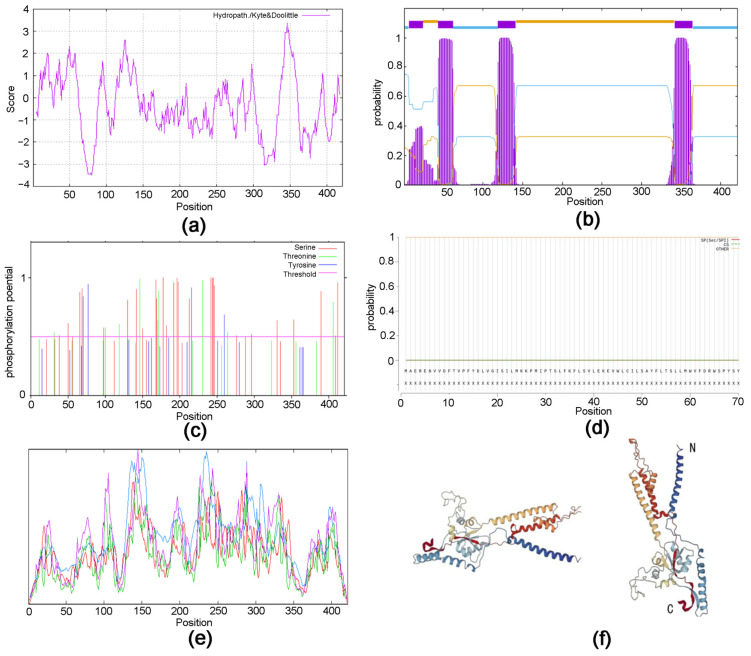
Protein structure prediction of EonuIR25a in *E. onukii*. (**a**) Hydrophilicity prediction of EonuIR25a protein. (**b**) Structure prediction of transmembrane region of EonuIR25a protein. (**c**) Phosphorylation site prediction of EonuIR25a protein. (**d**) Signal peptide prediction of EonuIR25a protein. (**e**) Secondary structure of IR25a protein in *E. onukii*. note: blue, red, green, and purple masks indicate alpha helix, extended strand, beta turn and random coil, respectively. (**f**) Tertiary structure of IR25a in *E. onukii*. Note: N and C indicate N- and C-termini of the protein.

The sequence obtained was identified by the National Center for Biotechnology Information (NCBI) as the *EonuIR25a* sequence with accession number OQ064772. The full-length open reading frame (ORF) consisted of 1266 nucleotides and encoded 422 amino acid residues; the molecular weight of the protein was 48.87 kD. The specificity of the gene was confirmed by agarose gel electrophoresis (Figure 1). The theoretical isoelectric point was 6.50, the extinction coefficient was 98,695, the lipid coefficient was 89.92, the instability coefficient was 55.03, the mean hydrophobicity coefficient was −0.372, and it is a hydrophilic protein (Figure 2a). The EonuIR25a protein was composed of 20 amino acids, with leucine as the main component, accounting for 10.2% of the total amino acids, and histidine accounting for only 1.0% of the total (Table S1). The amino acid sequence of the EonuIR25a protein has four transmembrane domains located at amino acids 7–24, 44–62, 119–141, and 342–364 (Figure 2b). The results of phosphorylation site prediction showed that EonuIR25a contains 46 phosphorylation sites, mainly in serine and threonine structures (Figure 2c), and no signal peptide in the amino acid sequence (Figure 2d). In the secondary structure of EonuIR25a, the α-helix is the main component of the amino acid sequence, accounting for 47.51% of the total structural proportion and corresponding to the transmembrane region, while the β-turn is lacking, accounting for only 3.09% of the total structural proportion (Figure 2e). The EonuIR25a contains the conserved structural domains of Lig_chan (positions 41–350) and SBP_bac_3 (positions 1–317), and the tertiary structure of EonuIR25a has similarity to the secondary structure, including the ligand-binding domain (LBD) (S1: 7–24, 119–141; S2: 44–62) and the ion channel domain, as well as the N-terminus on the outside of the cell membrane and the C-terminus on the inside (Figure 2f).

### 2.2. Multiple Sequence Alignment and Phylogenetic Tree Analysis

**Figure 3:**
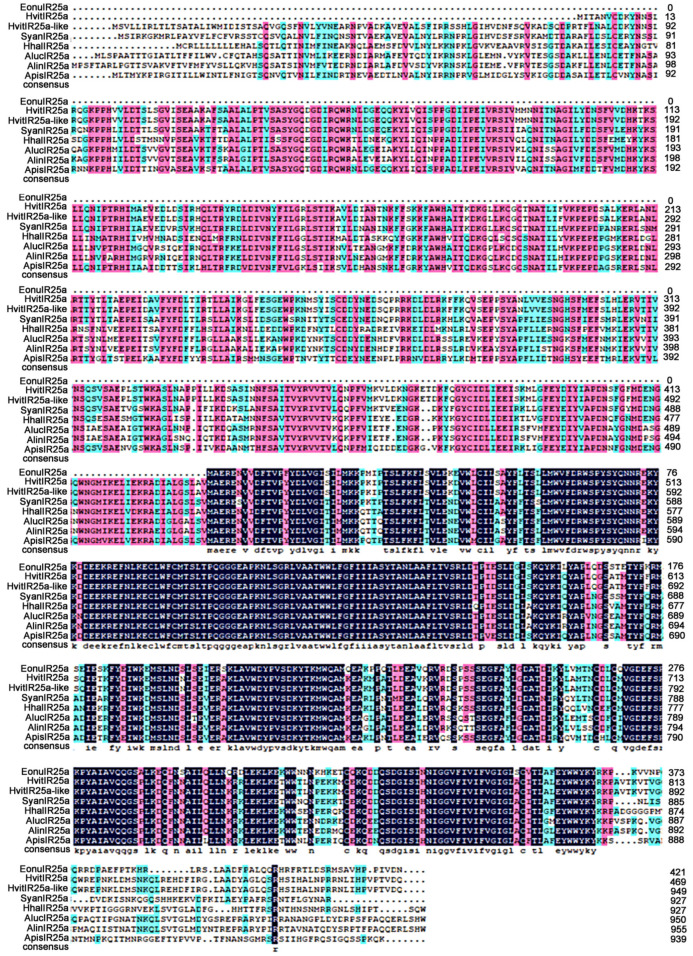
Amino acid sequence alignment of *EonuIR25a* with other hemiptera IRs. Note: *HvitIR25a*, *HvitIR25a-like*, *Homalodisca vitripennis* (KAG8282323.1, XP_046658708.1); *SyanIR25a*, *Subpsaltria yangi* (AXY87920.1); *HhalIR25a*, *Halyomorpha halys* (XP_024219750.1); *AlucIR25a*, *Apolygus lucorum* (QFU27937.1); *AlinIR25a*, *Adelphocoris lineolatus* (APZ81419.1); *ApisIR25a*, *Acyrthosiphon pisum* (XP_008183092.2). Black areas indicate 100% similarity, pink areas indicate more than 75% similarity, blue areas indicate more than 50% similarity.

**Figure 4:**
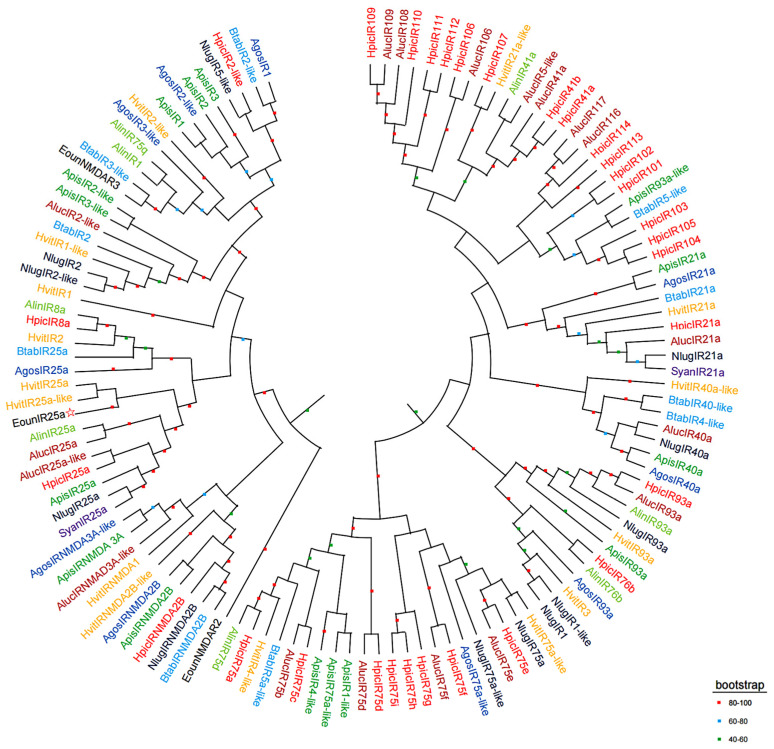
Phylogenetic tree of *EonuIR25a* with other hemiptera IRs based on amino acid sequences. Note: different species in the evolutionary tree are indicated by different colors. (Black) *Empoasca onukii*; (Red) *Halyomorpha halys*; (Purple) *Subpsaltria yangi*; (Yellow) *Homalodisca vitripennis*; (Brown) *Apolygus lucorum*; (Aquamarine) *Adelphocoris lineolatus*; (Blue) *Bemisia tabaci*; (Blue black) *Nilaparvata lugens*; (Navy blue) *Aphis gossypii*; (Green) *Acyrthosiphon pisum*. The target gene is indicated by a pentagram☆.

The results of multiple sequence comparison showed that the sequence similarity between *EonuIR25a* and IR25a of other hemiptera ranged from 30–40%, and the sequence similarity with *HvitIR25a* (KAG8282323.1) of *Homalodisca vitripennis* was relatively high, with a sequence similarity of 36.52%, while it has a low sequence similarity of 31.63% with *AlucIR25a* (QFU27937.1) of *Apolygus lucorum*. In addition, the similar sequences were mainly concentrated in the central and posterior parts (Figure 3). Phylogenetic tree analysis showed that *EonuIR25a* clustered into clearly separated branches, and it clustered with the IR25a of *Adelphocoris lineolatus*, *Apolygus lucorum*, *Halyomorpha halys*, *Nilaparvata lugens*, *Aphis gossypii*, and *Subpsaltria yangi*. *EonuIR25a* was clustered into the smallest branch with *HvitIR25a* and *HvitIR25a-like* of *Homalodisca vitripennis.* Within the IR25a branch, the sequences cluster in an order-specific manner, reflecting that the highest similarity exists between sequences of insects belonging to the same type of insect gene. On considering the highly conserved nature of IR25a, it can be speculated that it performs similar functions in different species (Figure 4).

### 2.3. Tissue Expression Analysis

**Figure 5:**
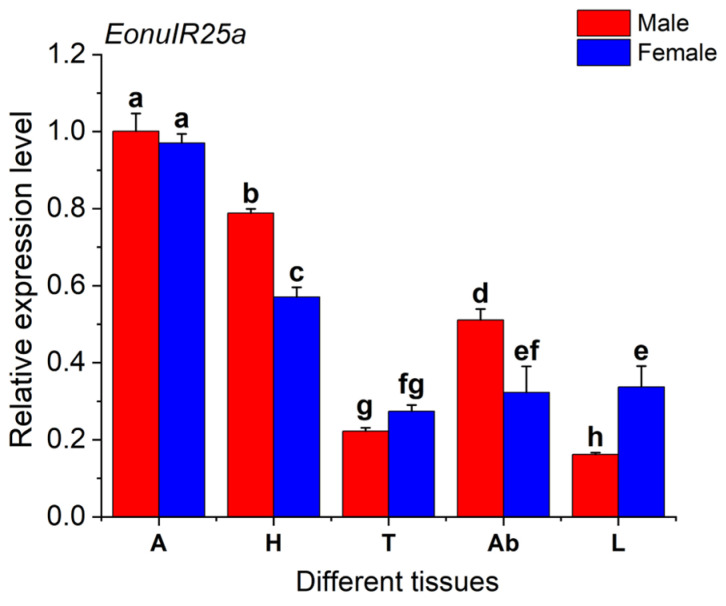
Relative expression levels of *EonuIR25a* in different tissues of *E. onukii*. Note: (A): antenna; (H): head; (T): thorax; (Ab): abdomen; (L): leg. β-actin was used in the expression analysis of different tissues of male and female adults *E. onukii* as a reference gene. Data in the figure are mean ± SE. Different lowercase letters on bars of the same color indicate significant differences in the relative expression levels of *EonuIR25a* among different tissues of male and female adults by Duncan’s test (*p* < 0.05).

The expression of *EonuIR25a* gene was 0.97 ± 0.01 and 1.00 ± 0.03 in female and male adult antennae, respectively, which was significantly higher than other tissues, and the expression of gene in male legs was only 0.16 ± 0.00. The expression of the *EonuIR25a* in male antennae was 6.16-fold and 4.5-fold higher than in male legs and male thorax, respectively. The distribution of *EonuIR25a* gene expression in the head and abdomen was higher in male adults than in females, but the distribution of expression in the legs was higher in female adults than in males. In addition, there was a significant difference between different tissues of *EonuIR25a* (*d.f.* = 9, *F* = 236.064, *p* < 0.01), and there was no difference in antennae and thorax between male and female adults of *EonuIR25a* (*p* > 0.05) (Figure 5).

### 2.4. Correspondence of the Compounds by E. onukii

**Figure 6:**
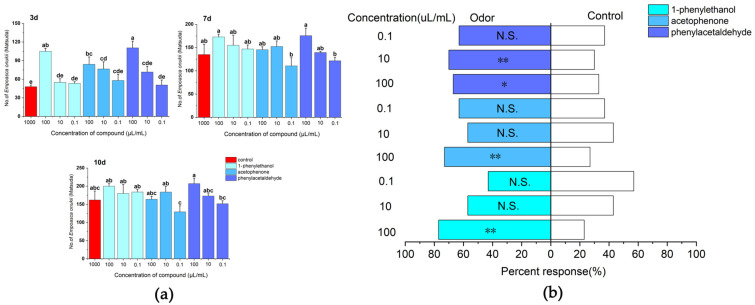
Responses of *E. onukii* to different concentrations of compounds in field trials and olfactometer bioassays. (**a**) Response of *E. onukii* to compounds in the field. (**b**) Response of *E. onukii* to different concentrations of compounds with paraffin in a Y-tube olfactometer. Note: asterisks indicate significant differences within a choice test (* *p* < 0.05; ** *p* < 0.01); N.S. indicates no significant difference; Different lowercase letters on bars indicate significant differences by Duncan’s test (*p* < 0.05).

After 3 days of treatment, phenylacetaldehyde was attractive to *E. onukii* at a concentration of 100 µL/mL, and the insect population of its sticky boards was 110.67 ± 10.7, with significant differences between the various treatments and the control (*d. f.* = 9, *F* = 7.392, *p* < 0.01). After 7 days of treatment, phenylacetaldehyde and 1-phenylethanol were attractive to *E. onukii* at a concentration of 100 µL/mL, with insect populations of 175.7 ± 16.2 and 173 ± 5.86, respectively, there was no difference in the attraction effect of the various treatments on *E. onukii* (*d. f.* = 9, *F* = 2.215, *p* > 0.05). After 10 days of treatment, 1-phenylethanol had the best attractive effects on *E. onukii*; the numbers of *E. onukii* trapped by 1-phenylethanol at a concentration of 100, 10, and 0.1 µL/mL were 200 ± 7, 180 ± 24.6, and 184 ± 6.6, respectively. There was significant difference in the attraction effect of the various treatments on *E. onukii* (*d. f.* = 9, *F* = 2.42, *p* = 0.048) (Figure 6a). On the Y-tube olfactometer tested, the *E. onukii* adults were more attracted to the volatiles including phenylacetaldehyde at a concentration of 10 µL/mL (*χ*^2^ = 7.251, *d. f.* = 1, *p* < 0.01), acetophenone at a concentration of 100 µL/mL (*χ*^2^ = 10.221, *d. f.* = 1, *p* < 0.01), and 1-phenylethanol at a concentration of 100 µL/mL (*χ*^2^ = 14.583, *d. f.* = 1, *p* < 0.01). There was a highly significant difference between these treatments and the control of attraction effect on *E. onukii.* The compounds 1-phenylethanol at a concentration of 0.1 µL/mL (*χ*^2^ = 0.724, *d. f.* = 1, *p* > 0.05), and acetophenone at a concentration of 10 µL/mL (*χ*^2^ = 0.724, *d. f.* = 1, *p* > 0.05) had low attractive effects on *E. onukii;* there was no difference between these treatments and the control of attraction effect on *E. onukii* (Figure 6b).

### 2.5. RNA Synthesis and Analysis of Interference Efficiency

**Figure 7:**
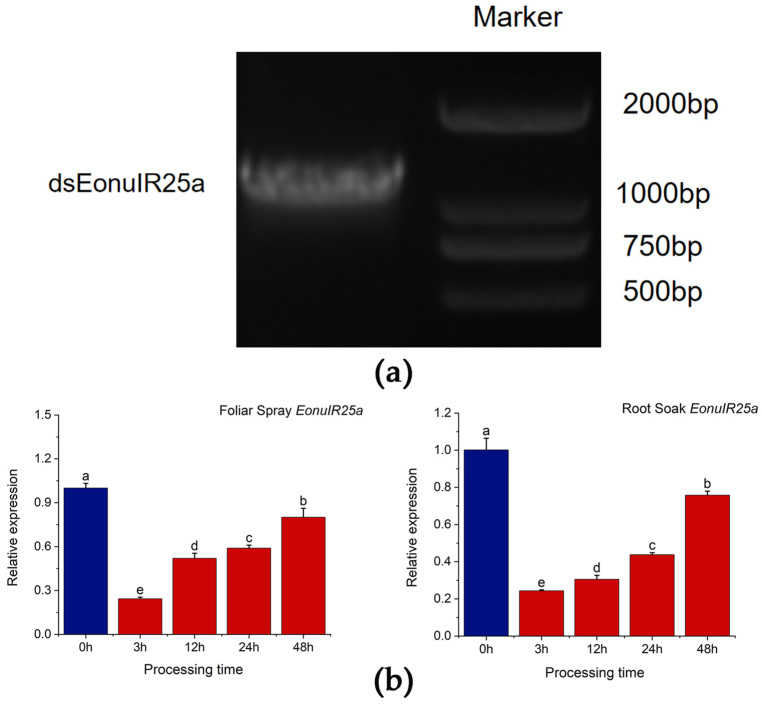
In vitro synthesis and RNA interference of *EonuIR25a*. (**a**) Identification of PCR products of dsEonuIR25a. (**b**) Relative expression of *EonuIR25a* was detected by root soak and foliar spray treatment for different time periods, respectively. Note: *β-actin* was used as a reference gene for the assay of *EonuIR25a* expression profiles in different tissues. Data in the graph are mean ± SE. Different lowercase letters on the color bar indicate that the relative expression levels of *EonuIR25a* were significantly different at various time periods by Duncan’s test (*p* < 0.05).

dsEonuIR25a was synthesized, and its accuracy was verified by 1% agarose gel electrophoresis; the product concentrations were tested to be above 2500 ng/µL, meeting the criteria for dsRNA to be used in subsequent experiments (Figure 7a). RNA interference in *E. onukii* was performed by feeding tea seedlings after root soaking and foliar spraying, while the interference efficiency of RNAi was measured by qRT-PCR to detect the expression of the *EonuIR25a* gene of *E. onukii* after different lengths of time. The qRT-PCR results showed that expression of *EonuIR25a* significantly decreased after 3 h of feeding on foliar spray treatment, and the gene expression decreased to 0.24 ± 0.01 at 3 h and 0.80 ± 0.35 at 48 h. The expression decreased by 75.67%, 48.04%, 41.07%, and 19.93% at 3, 12, 24, and 48 h after the foliar spray treatment, respectively; there was significant difference in expression between different duration of RNAi treatments and control (*p* = 0.03). The gene expression of *EonuIR25a* by root soaking was 0.24 ± 0.00 for 3 h and 0.76 ± 0.02 for 48 h. The expression of *EonuIR25a* decreased by 75.63%, 69.46%, 56.27%, and 24.21% at four time points after root soaking treatment, respectively; there was significant difference in expression between different duration of RNAi treatments and control (*p* = 0.03) (Figure 7b).

### 2.6. Changes in the Response of E. onukii to Compounds after Silencing EonuIR25a

**Figure 8:**
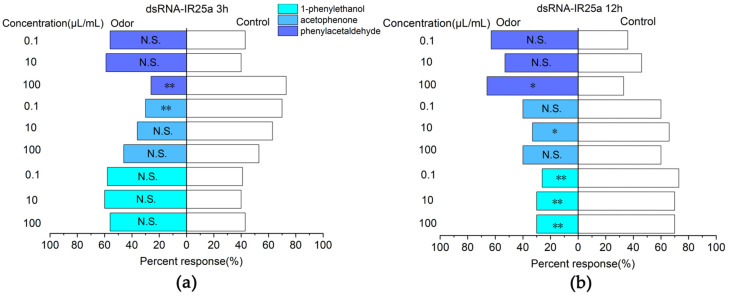
Responses of *E. onukii* to compounds versus paraffin in a Y-tube olfactometer. (**a**) Response rate of *E. onukii* to compounds after 3 h of interference with *EonuIR25a*. (**b**) Response rate of *E. onukii* to compounds after 12 h of interference with *EonuIR25a*. Note: asterisks indicate significant differences within a choice test (* *p* < 0.05; ** *p* < 0.01); N.S. indicates no significant difference.

The analysis of the efficiency of RNAi showed that the *E. onukii* fed tea seedlings treated by the root sock were more efficient in RNAi efficiency. Therefore, *E. onukii* was silenced with the *EonuIR25a* gene for 3 and 12 h, respectively, and was selected for Y-type olfactometer experiments. For 3 h, the responses to phenylethyl-aldehyde at a concentration of 100 µL/mL (*χ*^2^ = 10.221, *d. f.* = 1, *p* < 0.01) and acetophenone at a concentration of 0.1 µL/mL (*χ*^2^ = 7.251, *d. f.* = 1, *p* < 0.01) were significantly downregulated, which was significantly different from the control compared to the non-RNAi *E. onukii* (Figure 8a). For 12 h, the response rates of *E. onukii* to 1-phenylethanol at concentrations of 0.1 µL/mL (*χ*^2^ = 10.221, *d. f.* = 1, *p* < 0.01), 10 µL/mL (*χ*^2^ = 0.08, *d. f.* = 1, *p* < 0.01), and 100 µL/mL (*χ*^2^ = 7.251, *d. f.* = 1, *p* < 0.01) were significantly reduced, and there were significant differences between the treatments and the control. There were no significant differences between phenylethyl-aldehyde at concentrations of 0.1 µL/mL (*χ*^2^ = 2.93, *d. f.* = 1, *p* > 0.05), 10 µL/mL (*χ*^2^ = 1.636, *d. f.* = 1, *p* > 0.05), acetophenone at concentrations of 100 µL/mL (*χ*^2^ = 1.636, *d. f.* = 1, *p* > 0.05), 0.1 µL/mL (*χ*^2^ = 1.636, *d. f.* = 1, *p* > 0.05) and the control (Figure 8b).

## 3. Discussion

In this study, we cloned the gene encoding the *EonuIR25a* protein in *E. onukii*. The CDS length of *EonuIR25a* was 1266 bp and it encoded a 48.87 kD protein. After predictive analysis, *EonuIR25a* was found to have four transmembrane domains, mainly consisting of α-helices and irregularly coiled-winding folding. The sequence was found to contain the conserved structural domains of Lig_chan (positions 41–350) and SBP_bac_3 (positions 1–317); the Lig_chan family includes four transmembrane regions of ionotropic glutamate receptors and NMDA receptors, consistent with the typical structure of ionotropic receptors [38,39]. We found that there was similarity between *EonuIR25a* and IR25a/IR25a-like sequences of some hemiptera, which sequence similarity ranging from 30–40%. *EonuIR25a* is closely related to *HvitIR25a* of *H. vitripennis* with a sequence similarity of 36.52%, and the sequence similarity between *Drosophila* IRs has been reported to be 10–70%, implying that IRs are functionally diverse in insects [8]. Evolutionary tree analysis revealed that *EonuIR25a* clustered into the same branch with *HvitIR25a* and *HvitIR25a-like*, indicating that IR25a is highly conserved in different insects and presumably plays similar functions in insects [40]. This study showed that *EonuIR25a* was mainly distributed in the antennae and head, with a small amount of expression in the thorax, abdomen, and legs, while it is speculated that *EonuIR25a* may belong to the olfactory IRs [18]. In addition, studies on *Anopheles gambiae* [26], *Schistocerca gregaria* [41], and *Mythimna separata* [24] also demonstrated that olfactory IRs were mainly expressed in the neurons of the tentacle cone sensor. In addition, previous studies have shown that IRs are widely expressed in organ tissues of insects, mainly in the antennae, labellum, anterior wing margin, and legs, which is consistent with the results of this study [18,42]. Previous studies have shown that *Drosophila* IRs can sense odor substances, including acetic acid, propionic acid, butyric acid, hexanoic acid, 2-oxovaleric acid, and phenylacetic acid [8,43,44,45]. It was found that *Drosophila* IR8a and IR64a are expressed in adult antennal vesicle neurons to perceive acidic tastes [46]. In *Drosophila*, IR25a and IR76b are necessary for odor-evoked electrophysiological responses to amines [47]. From the tissue distribution, it was speculated that *EonuIR25a* may be involved in the perception of odorant substances by leafhoppers.

Plant volatiles help herbivorous insects locate hosts, and therefore, they could be used to help develop pesticide-free pest management strategies [48]. Compared with phenylacetaldehyde and acetophenone, the results of field and indoor tests showed that 1-phenylethanol had more attractive effects on *E. onukii*, indicating that alcohols have an effect on the behavioral choices of insects. Research indicated that a mixture of 1-phenylethanol had a significant attractive effect on 1st instar larvae of *Helicoverpa armigera* [49]. In addition, research showed that alcohols can attract insects such as *Dendroctonus frontalis* [50], *Helicoverpa assulta* [51], and *Dasychira baibarana* [52], which is similar to the results of this study.

The results of the Y-tube olfactometer showed that the response rate of *E. onukii* to 1- phenylethanol at concentration of 100 µL/mL was 56.67% after interfering with *EonuIR25a* for 3 h, which was not significantly different from the control. After 12 h of interference with *EonuIR25a*, the response rate to 1-phenylethanol at concentrations of 0.1, 10, and 100 µL/mL decreased to 26.67%, 30%, and 30%, respectively. The results of RNAi showed that the tendency of behavioral selection of *E. onukii* to 1-phenylethanol was reduced after interference with *EonuIR25a*, and it was speculated that *EonuIR25a* could function in the recognition of tea plant volatile 1-phenylethanol by *E. onukii*. The current functions for ionotropic receptor recognition of olfaction are less available and are mainly concentrated in model-living *Drosophila*. The ionotropic receptors of *Drosophila* can recognize alcohols such as *n*-propyl alcohol and ethyl alcohol [8]. In *Agrotis segetum*, *AsegIR75p.1* elicits electrophysiological signaling responses for alcohol ligand C6 unsaturated compounds [53], which is consistent with the results of the present research.

In recent years, RNAi has attracted much interest as a pest control tool [54], and the stability of dsRNA and the ultimate effectiveness and specificity of RNAi are worth considering [55]. Currently, the most commonly applied dsRNA delivery methods in insects are microinjection and feeding [56,57,58,59]. It is noteworthy that most microinjections are time-consuming and require a variety of equipment from in-house manufactured devices to complex microprocessor-controlled syringes, while microinjections are obviously limited to the laboratory [57]. In this study, the feeding method was chosen for RNAi due to the small size of *E. onukii*, and the high mechanical damage and mortality of microinjection found in the pre-experiment. *E. onukii* was found to exhibit olfactory defects in the recognition of 1-phenylethanol by feeding dsRNA, suggesting that this method could to some extent interfere with the target gene in *E. onukii*. The interference efficiency of root soaking was significantly more effective and stable than foliar spraying. It is assumed that dsRNA is absorbed into the vascular bundles of the plant through the roots and stays for a longer time, thus producing a stable interference effect. Meanwhile, considering the leaf area of tea seedlings, the dose of dsRNA and the number of sprays also had effects on the inhibitory effect of target genes, and our study tentatively verified that the feeding method was effective in interfering with *E. onukii* [60].

The method of feeding used in our study has the advantages of dsRNA being formulated into a sprayable form for application to large crops, thereby selectively targeting insects as they feed. In addition, feeding of dsRNA poses a moderate risk of resistance to gene silencing, while it can be targeted to appropriate transcripts with high fidelity without affecting nontarget species [58]. In particular, it should be noted that dsRNA is effective against insects that suck or chew on their mouthparts [61,62,63]. Therefore, feeding of dsRNA is not laborious, is easy to perform, and is applicable to insect pest management [64]. RNA biopesticides based on specific dsRNA have been developed and the safety of dsRNA for humans is also worth considering [65,66]. Currently, the risk of RNA biopesticides is assessed to be defined as safe by the European Food Safety Authority (EFSA), because the risk to animals and humans from sprayed dsRNA is very low [67,68]. The Organization for Economic Co-operation and Development (OECD) meeting concluded that dsRNA, as a nucleic acid material, has the same gene sequence composition as ingested in humans and other organisms. Nucleic acids are natural components of plant and animal food and feed and are daily consumables for humans and animals. At the same time, there are significant physiological and biochemical barriers in humans and other vertebrates, such as nucleases in saliva and the digestive tract, pH differences in gastric juices, and lysosomes in cells, which affect the uptake of exogenous dsRNA nucleic acids. Therefore, dsRNA is relatively safe for human health [69].

Olfactory signaling is a target for pest management, with attempts to interfere with and thus disrupt odorant- or pheromone-driven behaviors. Previous studies have shown that insect ionotropic receptors are involved in the olfactory recognition process [70,71]. The functions of several IRs in insects have been identified [70,71,72,73], but the molecular mechanisms and functions of IRs in most insects, especially in non-model insects, have not been reported. Therefore, there is an urgent need for functional studies of IRs in non-model insects. In summary, the field results indicate that 1-phenylethanol has an attractive effect on *E. onukii*, suggesting that the compound could produce behavioral modulation of *E. onukii*. The recognition ability of 1-phenylethanol in *E. onukii* was reduced after interference of *EonuIR25a* by RNAi, which indicates that *EonuIR25a* functions in the recognition of 1-phenylethanol by *E. onukii*, and it also reflects that there is certain plasticity in the olfactory mechanism of insects. In addition, the findings demonstrate that RNAi could be applied as an important tool for gene function verification. IRs could participate in the olfactory recognition process of insects; further experimental verification is needed regarding the other functions of IRs.

## 4. Materials and Methods

### 4.1. Insect Culture

Adults of *E. onukii* were collected from an organic tea garden at Shandong Taishan Chaxi Valley Agricultural Development Co., Tai’an, Shandong Province, China, in 2022. The colonies were kept and maintained on fresh tea seedlings in an artificial climate chamber in the laboratory of Shandong Agricultural University. The colony was maintained at 25 ± 1 °C, 60 ± 5% relative humidity, and a 16 h:8 h (L:D) photoperiod.

### 4.2. Total RNA Isolation and RT-PCR

Total RNA was isolated using the FastPure^®^ Cell/Tissue Total RNA Isolation Kit (Vazyme, Nanjing, China) from *E. onukii* adults. RNA samples were prepared and stored at −70 °C. RNA concentration and purity were assessed spectrophotometrically by measuring their absorbances at 260 and 280 nm in a biophotometer (Eppendorf, Germany). Gene-specific primers were designed to clone the ORF of the *EonuIR25a* gene, and cDNAs were synthesized from 2 l g of female antennal RNA using the MonScriptTm RTIIl All-in-One Mix (Mona, Wuhan, China). PCRs were conducted using Phanta Max Super-Fidelity DNA Polymerase (Vazyme, Nanjing, China) under the following conditions: denaturation at 95 °C for 3 min, 35 cycles of denaturation at 95 °C for 15 s, annealing at 55 °C for 55 s and extension at 72 °C for 1 min. The final extension step was at 72 °C for 5 min.

### 4.3. Cloning and Nucleotide Sequencing

The PCR products were then purified using the FastPure EndoFree Plasmid Mini Kit (Vazyme, Nanjing, China) and ligated into a *pET-30a* vector (Abiowell, Changsha, China) according to the manufacturer’s instructions. Afterward, plasmids were transformed into competent Escherichia coli DH5α competent cells, 100 μL of nonresistant LB solution was added, and the cells were shaken for 2 h in a 37 °C incubator and coated. After incubation in an incubator at 37 °C for 12 h, 20 positive clones of single bacteria were selected and incubated overnight at 37 °C in LB liquid medium containing 100 mg/mL Kana for PCR identification of the bacterial solution; the primers for PCR identification are shown in Table 1. The bacterial solution was identified as a 10 μL PCR system: 2 × Taq Master Mix 5 μL, 10 μmol/L forward, and reverse primers 0.4 μL each, ddH_2_O 3.2 μL, and bacterial solution 1 μL. Reaction conditions: denaturation at 95 °C for 3 min, 35 cycles of denaturation at 95 °C for 15 s, annealing at 55 °C for 15 s, and extension at 72 °C for 1 min. The final extension step was at 72 °C for 5 min. Several of these positive colonies were then purified using the FastPure^®^ Plasmid Mini Kit (Vazyme, Nanjing, China) and sent to Sangon Biotech Co., Ltd. (Shanghai, China) for sequencing.

### 4.4. Bioinformatics Analysis

The putative signal peptide was predicted using SignalP 3.0 Server (https://services.healthtech.dtu.dk/service.php?SignalP-5.0 (accessed on 15 October 2022)). The calculated molecular weight, theoretical isoelectric point, and grand average of hydropathicity were predicted using the ProtParm Tool (https://web.expasy.org/protparam/ (accessed on 15 October 2022)). The potential protein subcellular localization and transmembrane domains were predicted using WoLF PSORT (https://wolfpsort.hgc.jp/ (accessed on 15 October 2022)) and TMHMM Server (version 2.0) (https://services.healthtech.dtu.dk/service.php?TMHMM-2.0 (accessed on 15 October 2022)), respectively. Sopma (https://npsa-prabi.ibcp.fr/cgi-bin/npsa_automat.plpage=npsa_sopma.html (accessed on 15 October 2022)) and Swiss-Model (https://swissmodel.expasy.org/interactive (accessed on 15 October 2022)) were used to predict the secondary and tertiary structures, respectively. Server (http://www.cbs.dtu.dk/services/NetPhos/ (accessed on 15 October 2022)) was used to predict the protein phosphorylation sites; Protscale (https://web.expasy.org/protscale/ (accessed on 15 October 2022)) was used for protein hydrophilicity or hydrophobicity analysis.

### 4.5. Phylogenetic Analysis

To explore the similarity of candidate IR25a sequences from different insect orders, a hemiptera-based phylogenetic analysis was performed, including the EonuIR25a protein sequences and orthologs in other species. Sequence data were analyzed using DNAMAN (version 5.2) and the BLAST program (http://blast.ncbi.nlm.nih.gov/Blast.cgi (accessed on 1 December 2022)). The neighbor-joining tree was constructed using the MEGA 5.0 program with a *p*-distance model and pairwise deletion gaps [74]. Bootstrapping was performed by resampling the amino acid positions of 1000 replicates.

### 4.6. Expression in Several Tissues Using qRT-PCR

The relative transcript abundance of the *EonuIR25a* gene in the female antennae, male antennae, and body tissues (heads, thoraxes, abdomens, legs) was determined by qRT-PCR. The reference gene β-actin was also used for normalization, the qRT-PCR primers for *EonuIR25a* and β-actin for tissue distribution are shown in Table 1. The expression of *EonuIR25a* in several tissues was estimated by qRT-PCR using an ABI Prism 7500 Fast Detection System and SYBR Green SuperReal PreMix Plus (TianGen, Beijing, China). Each reaction was performed in a 20 μL final volume containing 10 μL 2 × ChamQ SYBR qPCR Master Mix, 0.4 μL each 10 μmol/L forward and reverse primers, 0.4 μL 50 × ROX Reference Dye 1, 1 μL template cDNA, and 7.8 μL ddH_2_O. The reactions were performed under the following conditions: pre-denaturation for 30 s at 95 °C, followed by 40 cycles of 10 s at 95 °C and annealing at 60 °C for 30 s, with a melting curve at 95 °C for 15 s, as instructed by the manufacturer. Each qRT-PCR experiment was performed with three biological replicates, and each biological replicate was assessed three times. The comparative 2^−ΔΔCt^ method [75] was used to calculate the relative transcript levels in each tissue.

### 4.7. Field Experiment

The experiment was conducted in September 2022 in the organic tea garden of Taishan Chaxi Valley Agricultural Co. The response of adult *E. onukii* to different doses of 3 volatile compounds was measured in the field using a combination of sticky boards and trap cores. The 3 volatile compounds were as follows: acetophenone (99%, Macklin, Shanghai, China), phenylacetaldehyde (95%, Macklin), and 1-phenylethanol (98%, Macklin). Solutions of each compound were prepared in paraffin at three concentrations (0.1 µL/mL: 1 µL of compounds mixed with 9999 µL of paraffin, 10 µL/mL: 1 µL of compounds mixed with 99 µL of paraffin, 100 µL/mL: 10 µL of compounds mixed with 90 µL of paraffin). A total of 750 µL of the volatile organic compound was added dropwise to a clean slow-release carrier, sealed in a bag, then placed in a refrigerator at 4 °C and used after the volatile organic compound was completely adsorbed by the slow-release carrier. For the field experiment, one trap core was fixed in the center of the sticky cards using wire as the treatment, with three replicates for each treatment, and paraffin was used as a blank control. The sticky cards were placed on tea trees in the ‘Fudingdabai’ variety park, and the treatments and replicates were distributed in a tessellated manner. The sticky cards were settled with an interval of 3 m. The numbers of *E. onukii* in each treatment were counted after 3, 7, and 10 d of the experiment.

### 4.8. Olfactometer Bioassays

The responses of *E. onukii* to tea plant volatiles were tested in a Y-tube olfactometer. Females and males of mixed ages used for olfactometer bioassays were collected from the artificial incubator which contained recently emerged *E. onukii* adults. The glass Y-tube olfactometer consisted of a 60-mm-long base tube and two 60-mm-long arms separated from each other at an angle of 90°. The inner diameter of the base tube and arms was 10 mm. Air was pumped into the apparatus by an electromagnetic air pump (ACO serial, Sunsun Group Co., LTD, China), filtered through activated charcoal and split into two air streams, each of which was fed through a glass flask and into one arm of the olfactometer at a speed of 60 mL/s controlled by a float-type flowmeter (LZB-3 WB, Changzhou Shuanghuan Thermo Technical Instrument Co., LTD, China). The two glass flasks (100 mL) provided the test and control odor sources. The connections between the components of the olfactometer were made of Teflon tubing. Olfactometer experiments were carried out in a dark room at 25 ± 2 °C and RH 60 ± 5%, and the observation time was 17:00–21:00 h. Test *E. onukii* were transferred individually to the base tube of the Y-tube and observed separately for 5 min. The choice of *E. onukii* for one of the two odor sources was recorded when it crossed a half-length of either arm within 5 min. If the tested *E. onukii* did not cross half the length of either arm after 5 min, it was recorded as ‘no choice’. After five *E. onukii* were tested, the odor sources entering the arms of the Y-tube were swapped to avoid directional effects due to lighting in the apparatus. Before each test, the apparatus was cleaned by rinsing with pure ethanol and dried in an oven (120 °C). The responses of adult *E. onukii* to acetophenone, phenylacetaldehyde, and 1-phenylethanol at different doses were measured using a Y-tube olfactometer. Solutions of each compound were prepared in paraffin at three concentrations (0.1, 10, and 100 µL/mL). One hundred microliters of the test solution was applied to a piece of filter paper (1 × 1 cm), which was placed into the treatment flask. Filter paper with 100 µL paraffin in the treatment flask was used as a control. Olfactometer bioassays were conducted as detailed above. The filter papers with the test solution and paraffin were changed every 20 min. Each odor comparison was repeated thirty times with one *E. onukii* each time.

### 4.9. DsRNA Synthesis

Using the *E. onukii* cDNA template and specific primers containing the T7 promoter sequence at their 5′ ends, regions of the E. onukii gene were amplified by qRT-PCR, and the primers are shown in Table 1. The profile used in the reactions included denaturation at 95 °C for 3 min, 35 cycles of denaturation at 95 °C for 15 s, annealing at 55 °C for 55 s, and extension at 72 °C for 1 min. The final extension step was at 72 °C for 5 min. PCR products were detected by 1% agarose gel electrophoresis and recovered using the Easy Pure Quick Gel Extraction Kit as a synthetic dsRNA template. Purified amplicons were transcribed in vitro to synthesize dsRNA using a T7 RNAi Transcription Kit (Vazyme, Nanjing, China). The integrity and quantity of dsRNAs were evaluated by spectroscopy analysis with a NanoDrop™ OneC Microvolume UV-Vis Spectrophotometer (Thermo Fisher Scientific) and by 1% agarose gel electrophoresis. The purified products were stored at −80 °C.

### 4.10. Feeding Method to Interfere with EonuIR25a in E. onukii

The prepared RNAi interference products were thawed, and the concentration was adjusted to 1000 ng/μL. Then, 400 μL of interference product was aspirated into a 1.5 mL enzyme-free centrifuge tube. The roots of an annual one-bud, three-leaf ‘Longjing 43′ tea seedling were cleaned with sterile water. The tea seedlings were air-dried until the root surface was free of droplet-like liquid and then immersed in enzyme-free centrifuge tubes for subsequent root sock treatment. The prepared RNAi interference fragment products were thawed, and the concentration was adjusted to 1000 ng/μL. One annual one-bud-three-leaf ‘Longjing 43′ tea seedling using sterile water was immersed in an enzyme-free centrifuge tube. dsEonuIR25a was sprayed onto the tea seedlings from four directions (approximately 400 μL) with a sprayer (2 mL) for subsequent foliar spray treatment. The tea seedlings were covered with 30 × 200 mm flat glass test tubes after root soaking and spray treatment. The mouth of the tube was sealed with a sealing film, and the sealing film was punctured with a 2 mm diameter hole by forceps. The test tubes were placed vertically upside down in a plant incubator, which was set to a temperature range of 25–28 °C, humidity of 45–50% rh, light intensity of 4000 LX, and photoperiod of 12 h. The samples were taken after 3 h, 12 h, 24 h, and 48 h of feeding on tea seedlings of *E. onukii*. Each of the 10 *E. onukii* were used as one sample, and the experiment had three biological replicates, which were collected and frozen in liquid nitrogen for use.

### 4.11. Data Statistics and Analysis

All statistical analyses were conducted with IBM-SPSS statistical software (v.18.0; IBM, Armonk, NY, USA). All qRT-PCR assays were performed using three biological replicates. The data were analyzed using the threshold cycle number (CT) and the 2^−∆∆Ct^ method, and significant differences in the expression patterns of the *EonuIR25a* gene in different tissues were analyzed using ANOVA followed by Duncan’s test. Significance was determined as *p* < 0.05. The indoor behavioral responses of the adult *E. onukii* in the olfactometer were analyzed using a *χ*^2^ test, and significance was determined as * *p* < 0.05; ** *p* < 0.01. The mean numbers of *E. onukii* in the field experiment were compared by Duncan’s test in ANOVA, and significance was determined as *p* < 0.05. Images were drawn by Origin64 software.

## Figures and Tables

**Table 1:** Primers of the target gene in *E. onukii*.

Gene	Forward Primer (5′–3′)	Reverse Primer (5′–3′)	PCR Type
*EonuIR25a*	GGGGTACCATGGCAGAGAGAGAGAATGTC	CCGCTCGAGTTAATTATCAACGATAGGCGGATG	RT–PCR
*EonuIR25a*	TCTTCAAGTTCCTCAGCGTTC	CATCTTTGTACTTCTCCCGATT	qRT–PCR
dsEonuIR25a	TAATACGACTCACTATAGGGATGGCAGAGAGAGAGAATGTC	TAATACGACTCACTATAGGGTTAATTATCAACGATAGGCGGATG	RT–PCR
β-actin	AGCGTGGTTACTCTTTCA	GCAACTCGTAGGACTTCT	qRT–PCR

## Data Availability

The raw data supporting the conclusions of this article will be made available by the authors, without undue reservation.

## Supplementary Materials

The following supporting information can be downloaded at: https://www.mdpi.com/article/10.3390/plants12102034/s1, Table S1: Amino acid composition of the EonuIR25a protein.
