# Supplementary material for: Characterization of Ionotropic Receptor Gene EonuIR25a in the Tea Green Leafhopper, Empoasca onukii Matsuda
Source: Plants (Basel). 2023 May 19;12(10):2034. doi: 10.3390/plants12102034 (PMC10223087; doi:10.3390/plants12102034)
Supplement: Supplementary file 1 [file plants-12-02034-s001.zip › plants-2356319-supplementary.pdf]

Supplementary Material

Characterization of Ionotropic Receptor Gene *EnouIR25a* in the Tea Green Leafhopper, *Empoasca onukii* Matsuda

Ruirui Zhang[1], Xiaoyue Lun[1], Yu Zhang[1], Yunhe Zhao[1], Xiuxiu Xu[2*], Zhengqun Zhang[1*]

*** Correspondence: Xiuxiu Xu[2*],yuayu88-66@163.com ;Zhengqun Zhang[1*],zqzhang@sdau.edu.cn**

1.Supplementary Tables

Table S1. Amino acid composition of the *EonuIR25a* protein

Amino acid	Number/Percentage (%)
Alanine (A)	6.2%
Arginine (R)	5.0%
Asparagine (N)	4.0%
Asparticacid (D)	5.7%
Cysteine (C)	1.4%
Glutamine (Q)	5.0%
Glutamicacid (E)	6.9%
Glycine (G)	3.8%
Histidine (H)	1.0%
Isoleucine (I)	5.2%
Leucine (L)	10.2%
Lysine (K)	7.4%
Methionine (M)	2.6%
Phenylalanine (F)	5.0%
Proline (P)	4.8%
Serine (S)	8.3%
Threonine (T)	4.5%
Tryptophan (W)	3.1%
Tyrosine (Y)	4.3%
Valine (V)	5.7%
